# Supplementary material for: Identification of the nuclear localization signal in the Saccharomyces cerevisiae Pif1 DNA helicase
Source: PLoS Genet. 2023 Jul 24;19(7):e1010853. doi: 10.1371/journal.pgen.1010853 (PMC10399864; doi:10.1371/journal.pgen.1010853)
Supplement: Attachment — Submitted filename: response to reviewers Lee et al final.pdf [file pgen.1010853.s008.pdf]

Point-by-point response to the Reviewer's and Editor's comments.

Responses to the Editor's comment.

1. Editor comment #1. PLOS policy is all data referred to in a manuscript must either be included in the manuscript/supplement or be publicly available and cited. This is the "no data not shown" rule. In the case of this manuscript, the Pif1-GFP localization data and the ChIP data in lines 134-141 are essentially "data not shown". Either these data must be included, or other experiments examining Pif1 localization should be done. In this regard, reviewer 1's comment about a bulky GFP adduct is germane. I think that the best course of action in addressing that comment and my comment 1 above would be to either use the Pif1-3xFLAG tagged construct, or to make a C-terminal fusion with an even smaller tag (we have had good results with a V5 tag) that will allow localization analysis either by immune-cytology or by subcellular fractionation. I cannot emphasize too much the importance of figuring out a way to directly measure the amount of Pif1 in the nucleus, and of showing that the KKRK mutant reduces this, and the SV40 NLS restores it". Further the Editor suggested: "I think it important to use a method more sensitive than live-cell GFP imaging to determine nuclear Pif1 levels…..including analysis of subcellular fractions on Western blots, ChIP-seq comparing the ratio of nuclear genome to mitochondrial genome reads, or ChIP PCR comparing nuclear and mitochondrial primer pairs."

Answer: As we mentioned in the original text, nuclear Pif1 is difficult to visualize. The abundance of Pif1 in the nucleus is very low because the vast majority of Pif1 protein is localized to mitochondria. We undertook three different attempts to detect Pif1 cytologically. First [1] work in the Zakian lab was unable to detect Pif1-GFP in the nucleus while it was readily detected in mitochondria; this work was published in Carly L. Geronimo's PhD thesis. Second, after receiving the reviewer's comments, the Malkova lab obtained previously published Pif1-YFP strains [2], but these also did not generate detectable nuclear signal. Third, the Malkova lab generated their own Pif1-RFP strain, but it also did not produce nuclear signal. Given that we could not detect nuclear WT Pif1 by three different cytological approaches in two different labs, clearly cytology is not sensitive enough to see reduced levels of nuclear Pif1 as expected for a strain where Pif1 lacks an NLS. (For the record, the Zakian lab easily saw *S, pombe* Pfh1-GFP in both mitochondria and nuclei, as shown on the cover of MCB [3]). Because PLoS Gen does not allow citing unpublished data, we removed all mention of cytological approaches from the revised manuscript.

Next, we used the Editor's suggestion to use ChIP analysis to compare directly the enrichment of Pif1 in WT vs *pif1* mutant strains at nuclear and mitochondrial locations. In particular, we analyzed the enrichment of Pif1 at two nuclear positions: *CEN13* (a known site of Pif1 binding [4]), and at *MAT* following BIR (where Pif1 is known to accumulate) [5]. At both sites, we observed 4-5x decrease in Pif1 enrichment in *pif1-NLS*Δ as compared to WT (see Fig. 7B in the revised manuscript). This reduction is in agreement with our genetic data (see Figs. 5, 6). We also used ChIP to show that Pif1 enrichment at a mitochondrial locus (position 31136bp based on yeast genome database) is the same in WT and *pif1-NLS*Δ cells (see new Fig. 7B). Thus, *pif1-NLS*Δ reduces Pif1 binding to its known nuclear targets but not its binding to mitochondrial DNA. In addition, binding of *pif1-NLSΔ+SV40* to nuclear and mitochondrial DNA was similar to WT (see Fig. 7B) suggesting that SV40 NLS compensates for the NLS deleted in *PIF1*.Together these data strongly support our hypothesis that ^{781}KKRK^{784} is a core of functional NLS.

Our ChIP data are described in a new Results section "**^{781}KKRK^{784} is important for Pif1 enrichment at nuclear, but not mitochondrial DNA binding sites**", and mentioned in the Discussion.

In addition, the following findings that were included into the revised version of this manuscript provide strong genetic support for the hypothesis that ^{781}KKRK^{784} is the core of a functional NLS. First, ^{781}KKRK^{784} sequence was identified by cNLS Mapper, a well-known software used to identify functional NLS sequences, and its sequence is typical for known NLS motifs: K-(K/R)-X-(K/R) [6]. Second, deletion of ^{781}KKRK^{784} reduces four known Pif1 nuclear functions: telomere lengthening, Okazaki fragment maturation, BIR efficiency, and Pif1 binding to nuclear targets while it does not influence mitochondrial Pif1 function. Third, all nuclear functions that were defective in *pif1-NLS*Δ are suppressed by addition of the SV40 NLS. Therefore, by both ChIP and highly sensitive genetic data we show that ^{781}KKRK^{784} is a functional NLS. Indeed, we believe that genetic tests are usually more stringent than cytological approaches. For example, our direct DNA synthesis BIR test (AMBER in Fig. 6) enabled us to capture a very small (less than 2-fold) difference between *pif1* mutant alleles.

2. Editor comment #2. The experiment shown in Figure 4 requires proper quantification and an internal control for transformation efficiency (for example, co-transformation with pRS316). The current snapshots of plate sectors are unacceptable as data.

Answer: We agree that the transformation assay that was used previously by us and others to establish that lack of Pif1 function suppresses *dna2* lethality is not a quantitative assay. Therefore, in the revised manuscript we present a new quantitative assay. In particular, we created two diploid strains with the following genotypes.

i) *DNA2/dna2*Δ::*URA3 pif1-NLS*Δ*/pif1*Δ::*KANMX*
ii) *DNA2/dna2*Δ::*URA3 PIF1/pif1*Δ::*KANMX*

Both strains are heterozygous for deletion of *DNA2* (*dna2*Δ::*URA3*) and *PIF1* (*pif1*Δ::*KANMX*). One of the strains also contained *pif1-NLS*Δ while the other one contained *PIF1* (WT). Following sporulation and dissection of 36 tetrads of the *DNA2/dna2*Δ::*URA3 PIF1/pif1*Δ::*KANMX* strain, there were 26 spores that were Ura^{+}G418^{r} (*dna2Δpif1Δ*)in the strain containing wild type *PIF1* (see new Fig. 8B), but, as expected, zero viable Ura^{+}G418^{s} (*dna2ΔPIF1*) spores. In contrast, when we dissected 35 tetrads of the *pif1-NLS*Δ*/pif1*Δ::*KANMX* strain, several (3 of 27) Ura^{+} spore clones were G418^{S} indicating that *dna2*Δ lethality can be partially suppressed by *pif1-NLS*Δ. As expected, in both diploids, there were an equal number of G418^{R} and G418^{S} spore clones among the Ura^{-} spores clones (see new Fig. 8B). The conclusion of partial suppression was further supported by random spore analysis of the same diploids (Fig. 8C). Together, the results of our quantitative assay supported our original conclusion that deleting ^{781}KKRK^{784} partially suppress the lethality of *dna2*Δ cells. However, as with our BIR data, these studies also show that *pif1-NLS*Δ is not a null allele. Our finding of partial suppression is consistent with small amounts of nuclear Pif1-NLSΔ (Figs. 5,6). These new findings are described in a new Results section, "**Partial suppression of *dna2Δ* lethality by *pif1-NLSΔ*"** and in the Discussion (see page 19, lines 405-410).

3. Editor comment #3. Details of yeast strains and plasmids need to be included. For example, pCG17 is described in Table S6 as pMB282 PIF1 with a C-terminal 3x FLAG tag, but pMB282 PIF1 is not described anywhere that I could find, and a contradictory description is provided in the Materials and Methods section. A proper description would include how many nucleotides of

the 5' and 3' UTRs are included, where in pRS414 the insert is located, etc., so that there is enough information to allow the experiments to be repeated. As another example, suppression of *dna2*Δ *l*ethality experiments are described as using plasmids transformed into YCG59, which is listed in the Materials and Methods as being pif1Δ dna2Δ but which is described in Table S1 as being a diploid that is heterozygous for *PIF1/pif1*Δ *DNA2/dna2*Δ.

Answer: Plasmid pRS414 [7] was used to clone plasmids used in this study. First, Pif1-3xFLAG was cloned into pRS414 under the control of *RRM3* promoter to create a plasmid pMB282 [8]. Second, the endogenous *PIF1* promoter was cloned into plasmid pMB282 digested by PspXI and AgeI to generate plasmid pCG17 [9]. Mutant alleles of *PIF1* were then introduced into pCG17 to generate pCG80 and pCG82. We also provided full genotypes of all yeast strains used in this work including YCG59 (see Materials and Methods, p. 21, "Yeast strains and growth conditions" and the Table S1).

The following description was added it the revised text:

p. 23, lines: 498-505: "pCG17 is a derivative of pRS414 that contains *PIF1* under its endogenic promoter and that was constructed in the following steps: (i) the PsPX1/AgeI fragment containing *PIF1* with a carboxy-terminal 3xFLAG tag under control of *RRM3* promoter was cloned into pRS414 digested with PsPX1 and AgeI to produce pMB282 [8]; (ii) the *RRM3* promoter in pMB282 was replaced with endogenous promoter of *PIF1* (using digestion with PsPX1 and AgeI) to create pCG17 [9]. pCG80 (see Table S6), which carries the NLSΔ allele, was generated from pCG17 by deleting sequence corresponding to amino acids ^{781}KKRK^{784} of Pif1."

Responses to Reviewer 1 comments.

4. Reviewer1 comment #1. The authors start by saying that Pif1 is too large to freely diffuse into the nucleus, so it must have an NLS. Then, their data indicate that some Pif1-NLSdelta must still make it to the nucleus, so perhaps diffusion does occur, or it is shuttled into the nucleus via interaction with another nuclear-targeted protein. Recent work has shown that there doesn't appear to be a hard barrier to diffusion based on molecular weight, with proteins as large as 230 kDa being able to diffuse into the yeast nucleus (J Cell Biol (2016) 215 (1): 57–76).

Answer: We thank the reviewer for pointing out this paper. The revised manuscript now cites it as support for our proposal that despite its size, some Pif1 may enter the nucleus passively. This possibility could explain at least in part how ^{781}KKRK^{784} can be the main NLS in Pif1, yet not be a null allele in all assays.

In response to this Reviewer's comment the following sentences were inserted into the revised Discussion:
p. 19, lines: 418-420 "Nuclear entry of Pif1 in the absence of the ^{781}KKRK^{784} motif:
"We envision at least three possibilities for how Pif1 enters the nucleus in *pif1-NLS*Δ cells. The most likely mechanism is that a fraction of the 98kDa Pif1-NLSΔ diffuses into the nucleus, as there is no sharp molecular weight cut off for passive protein import [10]."

5. Reviewer1 comment #2. It's clear that the NLSdelta mutant has nuclear defects, but could they be due to disrupting interaction with a protein binding partner that's common to telomere

maintenance, Okazaki fragment processing, and BIR? Pol delta is a possibility or even PCNA. The NLSdelta mutation affects the disordered CTD, and disordered regions of proteins are often used for protein-protein interactions. Have the authors investigated the Pif1 vs. Pif1-NLSdelta proteomes or purified Pif1-NLSdelta and used it in in vitro Okazaki fragment processing or BIR assays containing Pol delta? Building on the above comment, was any attempt made to verify that the biochemical activities of Pif1-NLSdelta are unchanged relative to WT Pif1? This helicase has been the subject of copious biochemical investigations, so it is strange that such a seemingly simple set of experiments was not attempted here.

Answer: In the revised manuscript, we note in the Discussion that we cannot exclude the possibility that deletion of ^{781}KKRK^{784} could disrupt an interaction between Pif1 and a protein binding partner that is simultaneously required for the Pif1 function in telomere maintenance, Okazaki fragment processing and BIR (but not in mitochondria). However, we also note that in our view this possibility is unlikely. Specifically, we are not aware of any known Pif1 binding proteins that will be required for all these three nuclear functions. For example, interaction between Pif1and Polδ could be required for Okazaki fragment procession and BIR [5], but it is unlikely involved in telomere maintenance, as inhibition of telomerase *in vitro* requires only Pif1 and telomerase [11]. In addition, as proposed by the reviewer, Pif1 does interact with PCNA, but this occurs via a non-canonical PCNA-binding site [12] (^{815}IAAMLQR^{831}) that is located distal to the ^{781}KKRK^{784} motif. Furthermore, our NLS hypothesis fits all of the criteria established in the literature for being an NLS. First, ^{781}KKRK^{784} is the consensus sequence for the core sequence of an NLS [6]. In contrast, as far as we can determine, there is no evidence that ^{781}KKRK^{784} is a protein-interaction motif for either Polδ, PCNA, or other key DNA metabolism factors. Parenthetically, the full NLS predicted by cNLS mapper in Pif1 is ^{777}DEQVKKRKLDY^{787}. We deleted just the core to minimize the risks of altering a function other than the NLS. Second, four of four nuclear functions are largely restored by attaching the SV40 NLS to Pif1 lacking ^{781}KKRK^{784}. In addition, the inserted SV40 NLS ^{126}PKKKRKV^{132} is flanked by amino acids that are different from the amino acids flanking the ^{781}KKRK^{784} motif in Pif1. Moreover, as shown in Fig. 1B, the SV40 motif is inserted 71 amino acids distal to the site of the removed NLS. Thus, the immediate amino acid context of any predicted protein-protein interaction site would be quite different in the SV40 NLS Pif1, which makes it unlikely that SV40-NLS restores a protein-protein interaction motif. Thus, although we cannot absolutely rule out that ^{781}KKRK^{784} interacts with a protein needed for nuclear functions (rather than for Pif1 import), we consider this an unlikely possibility.

We did not perform any proteomics or *in vitro* experiments on Pif1-NLSΔ. We believe that these experiments are well beyond the scope of this paper. We strongly disagree with the reviewer's description of the biochemistry as "simple" experiments. For example, in addition to Pif1 and Pif1-K264A, the assay for the role of Pif1 in Okazaki fragment maturation requires purification of Pol δ, Dna2, DNA ligase I, RFC, PCNA, RPA, and FEN1 (several of these are multi-subunits where each subunit has to be purified separately) [13]. The *in vitro* BIR assay uses purified Polδ, RFC, Pol32, Rad51, Rad54, RPA, and PCNA [5]. (Parenthetically, we and others – see for example, work from Kevin Raney's lab [14]– have done proteomics on Pif1 and found no candidates for an interacting protein that might be needed in the many diverse functions of nuclear Pif1). Taken together with what we think are persuasive data that indicate that the sequence we identified is an NLS, as established by earlier work from other labs, we feel that proteomics and biochemical experiments are well beyond the scope of this paper, especially as

this paper is being considered for publication in a journal that emphasizes and appreciates genetic approaches.

6. Reviewer1 comment #3. In lines 134-138, the authors state that they made Pif1-GFP fusions to investigate the sub-cellular localization of Pif1 mutants in a previous publication [9]. There is no mention of Pif1-GFP in that main text or its supplement, so perhaps the wrong paper is being cited? Regardless, the authors then state that Pif1-GFP was largely found in the mitochondria but not the nucleus. Assuming that the GFP is a C-terminal tag, it would be closer to the putative NLS than the N-terminal mitochondrial targeting sequence, so is it possible that a bulky C-terminal tag simply inhibits nuclear import? As-yet unpublished ChIP data are cited to bolster the notion that most Pif1 is mostly mitochondrial, but it is difficult to review this section lacking access to those data and methods. Did the authors try fixed cell imaging of Pif1 with a native antibody, an N-terminal tag, and/or a smaller C-terminal tag?

Answer: We apologize for the incorrect reference. Our attempts to use a cytological approach to determine the localization of Pif1-NLSΔ are described in our answer to the Editor's comment 1 (see above). Briefly, using three different tagging strategies in two different labs, we are unable to detect nuclear Pif1, while mitochondrial Pif1 is readily detected. (For comparison, we readily detect the *S.pombe* Pfh1-GFP in both nuclei and mitochondria [3], the cover of that issue shows the localization). As we noted in the original manuscript, genome-wide ChIP-seq shows that the vast majority of Pif1 is in mitochondria. As these data are not published, to conform with PLoS Genetics rules, we do not mention them in the revised text. Rather, we carried out new locus-specific ChIP experiments for this paper. As anticipated if ^{781}KKRK^{784} is the core of an NLS, Pif1-NLSΔ binding to two different nuclear sites is reduced 4-5-fold as compared to WT Pif1 whereas its binding to a mitochondrial site is similar to WT Pif1 (see Fig. 7B and answer #1 for details). In addition, we believe that the results of our genetic approaches provide the most sensitive and strong support for our conclusion of "^{781}KKRK^{784}" being a functional NLS (see answer #1 for details).

7. Reviewer1 comment #4. In line 167, Pif1 should not be italicized (or should be in all capital letters in italics). In the same paragraph, the authors state that a role of Pif1 is "unwinding the newly synthesized in a D-loop DNA from its template". The newly synthesized what? Is "DNA" just misplaced in that sentence?

Answer: Thank you. These errors are corrected in the revised text.

8. Reviewer1 comment #5. In the Discussion, the fact that the pif1-m2, pif1-NLSdelta, and pif1-m2+NLSdelta are not nuclear nulls is brought up again. Residual nuclear Pif1 is then hypothesized to be due to translation starting from the third AUG of the PIF1 ORF. Assuming the same reading frame, that isoform would still lack an NLS in the NLSdelta alleles though. How much smaller is that isoform relative to pif1-m2; would it be expected to freely diffuse into the nucleus much faster? Regardless, why not try to make this hypothetical pif1-m3 mutant to assess its sub-cellular localization. Alternatively, one could fuse a nuclear export sequence onto pif1-m2, pif1-NLSdelta, and/or pif1-m2+NLSdelta to help eliminate residual Pif1 from the nucleus.

Answer: We eliminated the possibility of Pif1 translation from the third AUG from the revised manuscript as a recent paper shows that the resulting protein would not be fully functional [15].

As noted above, we propose that the small amount of nuclear Pif1 in *pif1-NLS*Δ cells is likely due to passive diffusion (see our answer #4 to the Reviewer 1 for details).

9. Reviewer1 comment #6. The Southern blot in Figure 3 is of poor quality. Perhaps this is due to it being a low-resolution file at upload, or maybe the contrast just needs to be adjusted. As is, it is difficult to see the telomere signal relative to the molecular weight markers.

Answer: In response to this comment, we redid the telomere blot and obtained the same conclusion. We believe this new Southern blot (see new Fig. 3) will satisfy the reviewer's concerns.

Responses to Reviewer 2 comments.

10. Reviewer2 comment #1. The experiments follow a logical progression, and the quality of data is high. However, I have two substantial criticisms of the manuscript. First, as detailed below, authors overstate the strength of their conclusions. Second, the description of the BIR experiments is difficult to follow. The basic issue is whether the authors have demonstrated that the KKRK sequence is the NLS. In the Introduction, they point out that large proteins enter the nucleus either because they have an NLS or because they interact with proteins that have an NLS. If the KKRK sequence is required for Pif1 to interact with an NLS-containing protein, their results would be the same as if KKRK was the NLS. The control of adding an NLS derived from SV40 does not really help their argument, since this sequence could substitute for the NLS provided by the mystery yeast protein. Of course, one can argue that the issue is somewhat semantic since the KKRK sequence may be acting as the NLS indirectly. However, the authors favor the more direct effect of KKRK.

Answer: We agree with this reviewer that we cannot fully exclude this possibility. Therefore, we included the following sentence into the Discussion section:

p. 18. Line 384-386: "We still cannot fully exclude that this motif is required for the interaction with another NLS-containing protein, but even if it is true, it would still support our overall conclusion that ^{781}KKRK^{784} is required for the nuclear import of Pif1."

11. Reviewer2 comment #2. I found the discussion of the effect of the KKRK on BIR difficult to follow. In part, this difficulty is because of Figure 5. More specifically, Fig. 5A does not show some of the events subsequently depicted in Fig. 5B. If I understand the system, the researchers distinguish four classes by replica-plating and colony color: Ade$^+$ Leu$^-$ (labeled BIR in Fig. 5A); Ade$^-$ Leu$^-$ White (labeled HC in Fig. 5A); Ade$^-$ Leu$^-$ Red (labeled CL in Fig. 5A); Ade$^+$ Leu$^-$ White (labeled BIR in Fig. 5A); Ade$^+$ Leu$^+$ (labeled GC in Fig. 5A). In Fig. 5B, two other terms are introduced, HC-II and GCR. It would help if these events were included in Fig. 5A.

Answer: In response to this reviewer's comment, we simplified the BIR part of the manuscript. In particular, our phenotype classes in the new Figure 4A (former Figure 5A) now agree with the DSB repair outcomes shown in the new Figure 4B (former Figure 5B). Also, the classes presented in the Figure 4B are now based only on visible phenotypes observed directly in experiment and do not include any "calculated" DSB outcomes. In the revised manuscript, the results section "**Pif1's role in break-induced replication (BIR) requires amino acids ^{781}KKRK^{784}**" now includes the results based only on directly observable phenotypes. All of the complex BIR outcomes (including all calculated outcomes resulting from failed BIR (such as

GCRs and HC-II)) are now presented separately in the Results section called **"BIR in *pif1-NLS*Δ leads to chromosomal rearrangements"** and in Figures 4C and 4D.

12. Reviewer2 comment #3. Another issue is that Lee et al. separated the Ade^+ Leu^- class into two sub-classes, BIR and GCR, using CHEF gels. Why not show these classes separated in Fig. 5B instead of having a single BIR + GCR class? A more substantial problem is the HC-II class. If I understand the description of this class, it is indistinguishable from the BIR class, containing one recombinant chromosome and one parental chromosome. Apparently, the frequency of HC-II was estimated by assuming that the frequency of HC-I was equal to that of HC-II. Why this assumption is reasonable is not discussed in the manuscript and needs to be. Unless the authors can give a strong argument that HC-I=HC-II, it would be better to simply present the distribution of events in the various strains into the phenotypic classes that they can distinguish.

Answer: The revised version of the manuscript now includes a separate section where we present the data on GCR (analyzed by CHEF gels) and on half-crossover II (HC-II) outcomes (named "**BIR in *pif1-NLS*Δ leads to chromosomal rearrangements")**. In addition, we included a new schematic to illustrate the formation of HC-II outcomes (See Fig. 4E for details). We hope this resolves this reviewer's concern. The data on GCR frequencies as well as our calculated data on HC-II are now presented on separate panels (Figs. 4 C and D). In addition, the assumption that the frequency of HC-I is equal to that of HC-II is described in our previous study [16] and we included this reference in the revised text. We also included the details on the HC-II calculation and on this assumption in the Results, Materials and Methods, and also in the legend to the Figure 4E. We hope these changes clarify the description of BIR results.

In response to this Reviewer's comment the following sentences were inserted into the Results section, Materials and Methods, and into the legend to Figure 4:

In Results (p. 10, lines: 208-217): "Moreover, we previously proposed [16] that many of even non-rearranged Ade^+Leu^- colonies in *pif1* mutants likely resulted not from completed BIR events, but from the second type of half-crossovers that we call HC-II (see Fig. 4D and schematics in Fig. 4E for explanation). In brief, HC-II events result from mitotic segregation of half-crossover chromosomes with an intact copy of the donor chromosome, which leads to the formation of Ade^+Leu^- colonies. Assuming that the frequencies of Ade^{-white} Leu^- half-crossover (HC-I) and of HC-II classes are equal [16], we can estimate the fraction of HC-II (invisible) class among Ade^+Leu^- events for each strain background. Following the subtraction of GCR and HC-II from Ade^+Leu^- events, it appears that real BIR events are rarely completed in all *pif1* mutants including *pif1-NLS*Δ, but successfully completed in *pif1-NLS*Δ*+SV40* (similarly to WT) (Fig. 4D)."

In Material methods (p. 27, lines: 591-595): "Due to random segregation of chromosomes, half of the HC products segregate with an intact copy of full-length chromosome III leading to formation of repair products (HC-II) that are genetically indistinguishable from BIR [16]. Therefore, it was assumed that the number of HC-II events is equal to the number of HC-I. Consequently, the number of BIR was adjusted by subtracting the number of HC-II."

In the legend to Figure 4 (p. 37, lines: 904-908): "The distribution of all repair DSB events, including calculated % of three classes contributing to the formation of Ade^+Leu^- outcomes (from B), including BIR, half-crossover II (HC-II), and GCRs. The amount of GCRs was

calculated by applying the fraction of GCR calculated from C to the total Ade^+Leu^- from B. The number of HC-II was assumed to be equal to HC-I based on B [16]."

13. Reviewer2 comment #3. As the manuscript is currently written, the authors may be over-estimating the effect of the KRKK deletion on BIR because they subtract their estimate of the HC-II class from the BIR+GCR class. If they use only the phenotypic class that they can detect, the effect of the KKRK deletion on BIR is considerably more modest.

Answer: Our revised version of Results makes the conclusion based only on the frequency of Ade^+Leu^- events that are directly scored (Fig. 4B). We also mention that the actual frequency of completed BIR in all strains would be lower after subtracting GCR and HC-II events (Fig. 4D and see also the text in the **"BIR in *pif1-NLS*Δ leads to chromosomal rearrangements"** section).

14. Reviewer2 comment #4. A relatively minor point is that the authors should add a few sentences describing the "AMBER" technique, and how it differs from standard Q-PCR. Does Fig. 7 indicate that the centromere-distal fragment of the "red" chromosome is degraded quickly following HO cutting? Alternatively, is the PCR targeted somehow to the "green" chromosome?

Answer: In response to this reviewer's comment, we added a brief description to the Results of the "AMBER" technique, and an explanation of how it differs from standard quantitative-PCR technique. We also note why it represents the best choice for assessing kinetics of BIR. In addition, we would like to answer that PCR is targeted specifically to the donor (green) chromosome. The centromere-distal fragment of the red chromosome has only 46bp of homology to the donor, and this homology is not targeted by PCR. In addition, the centromere-distal fragment (red) is degraded approximately 2 hours after the DSB [17].

In response to this Reviewer's comment the following sentences were inserted into the Results section: <u>(p. 12, lines 253-263):</u> "Therefore, we used the digital droplet PCR (ddPCR) based on AMBER (<u>A</u>ssay for <u>m</u>onitoring <u>B</u>IR <u>e</u>longation <u>r</u>ate) assay, which precisely measures the amount of DNA synthesized during individual BIR events [18, 19] by monitoring copy number changes at different BIR positions and at multiple time points after galactose addition (Fig. 6A, see legend for details). AMBER uses **donor-specific primer** sets for three separate locations within a 61 kb BIR region and then normalizes these values to the signals obtained at the *ACT1* locus to determine relative copy number."

15. Reviewer2 comment #5. In summary, Lee et al. have presented a detailed analysis of the effects of the KKRK deletion on a variety of nuclear phenotypes. In my view, the conclusions are over-stated and need to be qualified. In addition, the effect of the mutation on some aspects of BIR is written in a confusing manner and needs to be clarified.

Answer: We hope the reviewer agrees that the revised manuscript addresses all of his/her concerns.

<u>Responses to Reviewer 3 comments.</u>

16. Reviewer3 comment #1. Pif1 helicases are multi-tasking helicases that have functions in the mitochondrial and nuclear genomes. Pif1 helicases are evolutionary conserved and the budding yeast Pif1 was the first described member of Pif1 family helicases and is the most studied one. Although Pif1 is not an essential gene in budding yeast, its deletion causes mitochondrial defects,

and is therefore not an optimal strain to use in genetic studies. Therefore, the pif1-m2 allele has been the best model so far. In this manuscript, the Zakian and Malkova labs identified a candidate motif for the nuclear localization signal in the C-terminus region of Pif1. They convincingly show by several genetic assay that the absence of this motif results in nuclear, but not mitochondrial defects, demonstrating that the motif signals for nuclear import of Pif1. The paper is very well-written, easy to follow, and the experiments are clear. I expect that this new finding and the constructed strain will be very useful and welcomed in many labs that study genomic stability.

Answer: We thank this reviewer for his/her very positive assessment of our work.

17. Reviewer3 comment #2. Table S1 – The genotype of the strains is not fully written out but each strain is referred to by a previous strain name and then “but x”. Please also indicate the full genotype of each strain as the genotypes of the different strain are not clear now.

Answer. The Table S1 was modified accordingly.

References.

1. Geronimo. CL. Ph.D. Dissertations "Mutational and functional analysis of the conserved Pif1 DNA helicase in *Saccharomyces cerevisiae*". Princeton University2019.
2. Wagner M, Price G, Rothstein R. The absence of Top3 reveals an interaction between the Sgs1 and Pif1 DNA helicases in *Saccharomyces cerevisiae*. Genetics. 2006;174(2):555-73. Epub 20060702. doi: 10.1534/genetics.104.036905. PubMed PMID: 16816432; PubMed Central PMCID: PMCPMC1602079.
3. Pinter SF, Aubert SD, Zakian VA. The *Schizosaccharomyces* pombe Pfh1p DNA helicase is essential for the maintenance of nuclear and mitochondrial DNA. Mol Cell Biol. 2008;28(21):6594-608. Epub 2008/08/30. doi: 10.1128/MCB.00191-08. PubMed PMID: 18725402; PubMed Central PMCID: PMCPMC2573227.
4. Chen CF, Pohl TJ, Pott S, Zakian VA. Two Pif1 Family DNA Helicases Cooperate in Centromere Replication and Segregation in *Saccharomyces cerevisiae*. Genetics. 2019;211(1):105-19. Epub 2018/11/18. doi: 10.1534/genetics.118.301710. PubMed PMID: 30442759; PubMed Central PMCID: PMCPMC6325707.
5. Wilson MA, Kwon Y, Xu Y, Chung WH, Chi P, Niu H, et al. Pif1 helicase and Poldelta promote recombination-coupled DNA synthesis via bubble migration. Nature. 2013;502(7471):393-6. Epub 2013/09/13. doi: 10.1038/nature12585. PubMed PMID: 24025768; PubMed Central PMCID: PMCPMC3915060.
6. Lange A, Mills RE, Lange CJ, Stewart M, Devine SE, Corbett AH. Classical nuclear localization signals: definition, function, and interaction with importin alpha. J Biol Chem. 2007;282(8):5101-5. Epub 2006/12/16. doi: 10.1074/jbc.R600026200. PubMed PMID: 17170104; PubMed Central PMCID: PMCPMC4502416.
7. Sikorski RS, Hieter P. A system of shuttle vectors and yeast host strains designed for efficient manipulation of DNA in *Saccharomyces cerevisiae*. Genetics. 1989;122(1):19-27. Epub 1989/05/01. doi: 10.1093/genetics/122.1.19. PubMed PMID: 2659436; PubMed Central PMCID: PMCPMC1203683.
8. Paeschke K, Bochman ML, Garcia PD, Cejka P, Friedman KL, Kowalczykowski SC, et al. Pif1 family helicases suppress genome instability at G-quadruplex motifs. Nature. 2013;497(7450):458-62. Epub 2013/05/10. doi: 10.1038/nature12149. PubMed PMID: 23657261; PubMed Central PMCID: PMCPMC3680789.

9. Geronimo CL, Singh SP, Galletto R, Zakian VA. The signature motif of the *Saccharomyces cerevisiae* Pif1 DNA helicase is essential *in vivo* for mitochondrial and nuclear functions and in vitro for ATPase activity. Nucleic Acids Res. 2018;46(16):8357-70. Epub 2018/09/22. doi: 10.1093/nar/gky655. PubMed PMID: 30239884; PubMed Central PMCID: PMCPMC6144861.
10. Timney BL, Raveh B, Mironska R, Trivedi JM, Kim SJ, Russel D, et al. Simple rules for passive diffusion through the nuclear pore complex. J Cell Biol. 2016;215(1):57-76. Epub 20161003. doi: 10.1083/jcb.201601004. PubMed PMID: 27697925; PubMed Central PMCID: PMCPMC5057280.
11. Boule JB, Vega LR, Zakian VA. The yeast Pif1p helicase removes telomerase from telomeric DNA. Nature. 2005;438(7064):57-61. Epub 2005/08/27. doi: 10.1038/nature04091. PubMed PMID: 16121131.
12. Buzovetsky O, Kwon Y, Pham NT, Kim C, Ira G, Sung P, et al. Role of the Pif1-PCNA Complex in Pol δ-Dependent Strand Displacement DNA Synthesis and Break-Induced Replication. Cell Rep. 2017;21(7):1707-14. doi: 10.1016/j.celrep.2017.10.079. PubMed PMID: 29141206; PubMed Central PMCID: PMCPMC5842794.
13. Ayyagari R, Gomes XV, Gordenin DA, Burgers PM. Okazaki fragment maturation in yeast. I. Distribution of functions between FEN1 AND DNA2. J Biol Chem. 2003;278(3):1618-25. Epub 20021106. doi: 10.1074/jbc.M209801200. PubMed PMID: 12424238.
14. Zybailov B, Gokulan K, Wiese J, Ramanagoudr-Bhojappa R, Byrd AK, Glazko G, et al. Analysis of Protein-protein Interaction Interface between Yeast Mitochondrial Proteins Rim1 and Pif1 Using Chemical Cross-linking Mass Spectrometry. J Proteomics Bioinform. 2015;8(11):243-52. Epub 20151119. doi: 10.4172/jpb.1000376. PubMed PMID: 26807012; PubMed Central PMCID: PMCPMC4722957.
15. Nickens DG, Sausen CW, Bochman ML. The Biochemical Activities of the *Saccharomyces cerevisiae* Pif1 helicase are regulated by its N-terminal domain. Genes (Basel). 2019;10(6). Epub 20190528. doi: 10.3390/genes10060411. PubMed PMID: 31142053; PubMed Central PMCID: PMCPMC6628001.
16. Deem A, Barker K, Vanhulle K, Downing B, Vayl A, Malkova A. Defective break-induced replication leads to half-crossovers in *Saccharomyces cerevisiae*. Genetics. 2008;179(4):1845-60. Epub 2008/08/09. doi: 10.1534/genetics.108.087940. PubMed PMID: 18689895; PubMed Central PMCID: PMCPMC2516063.
17. Malkova A, Naylor ML, Yamaguchi M, Ira G, Haber JE. *RAD51*-dependent break-induced replication differs in kinetics and checkpoint responses from *RAD51*-mediated gene conversion. Mol Cell Biol. 2005;25(3):933-44. doi: 10.1128/MCB.25.3.933-944.2005. PubMed PMID: 15657422; PubMed Central PMCID: PMCPMC544012.
18. Liu L, Sugawara N, Malkova A, Haber JE. Determining the kinetics of break-induced replication (BIR) by the assay for monitoring BIR elongation rate (AMBER). Methods Enzymol. 2021;661:139-54. Epub 20211029. doi: 10.1016/bs.mie.2021.09.004. PubMed PMID: 34776210.
19. Liu L, Yan Z, Osia BA, Twarowski J, Sun L, Kramara J, et al. Tracking break-induced replication shows that it stalls at roadblocks. Nature. 2021;590(7847):655-9. Epub 2021/01/22. doi: 10.1038/s41586-020-03172-w. PubMed PMID: 33473214; PubMed Central PMCID: PMCPMC8219245.
