# Supplementary material for: Identification of the nuclear localization signal in the Saccharomyces cerevisiae Pif1 DNA helicase
Source: PLoS Genet. 2023 Jul 24;19(7):e1010853. doi: 10.1371/journal.pgen.1010853 (PMC10399864; doi:10.1371/journal.pgen.1010853)
Supplement: Attachment — Submitted filename: response to reviewers 0628.docx [file pgen.1010853.s009.docx]

***Point-by-point response to the Reviewer’s and Editor’s comments.***

**Responses to the Editor’s comment.**

1. **Editor comment #1.** There is one small data availability issue that still needs to be addressed, regarding the data is Supplemental Table 3, relevant to Figure 5. This table reports rates of Lys+ mutants as median values of a number of repeats. PLOS data availability policy mandates reporting underlying numerical data, which in this case I think are the individual frequencies of Lys+. Please modify supplemental Table 3 so that underneath the summary table (which is excellent, please keep it!), you report the individual values (i.e. Lys+ frequency at 0h and Lys+ frequency at 7h) for each repeat, i.e. for WT, before and after galactose values for repeat 1, repeat 2,...repeat 9.
   **Answer:** In response to this comment we decided to make an additional Supplemental Table (new Supplemental Table 7), which contains all information about each individual experiment used for the calculation of frequencies shown in Supplemental Table 3 including all individual frequencies, rates and number of Lys^+^ colonies in each individual experiment. It is our hope that this information is sufficient.
2. **Reviewer 2 comment #1.**

To describe the type of statistical method used in Figure 7B and the p-values for *

**Answer:** Asterisks represent statistically significant difference as determined by unpaired t-test (***: p=0.0005, *:p<0.05). This sentence was inserted in the legend to Figure 7B.

3. **Reviewer 2 comment #2.** To correct a typo that was found on page 8 on line 145, "carbon course" should be changed to "carbon source".

**Answer:** corrected.
